# Comparative Study of Single Crystal and Polymeric Pyroelectric Detectors in the 0.9–2.0 THz Range Using Monochromatic Laser Radiation of the NovoFEL

**DOI:** 10.3390/polym15204124

**Published:** 2023-10-18

**Authors:** Anatoly R. Melnikov, Evgeny V. Kalneus, Yaroslav V. Getmanov, Darya A. Shevchenko, Vasily V. Gerasimov, Oleg A. Anisimov, Matvey V. Fedin, Sergey L. Veber

**Affiliations:** 1 International Tomography Center, Siberian Branch of the Russian Academy of Sciences, 3a, Institutskaya Str., 630090 Novosibirsk, Russia; mfedin@tomo.nsc.ru; 2 Novosibirsk State University, 1, Pirogova Str., 630090 Novosibirsk, Russia; kalneus@kinetics.nsc.ru (E.V.K.); y_getmanov@mail.ru (Y.V.G.); v.v.gerasimov3@gmail.com (V.V.G.); 3 Voevodsky Institute of Chemical Kinetics and Combustion, Siberian Branch of the Russian Academy of Sciences, 3, Institutskaya Str., 630090 Novosibirsk, Russia; anisimov@academ.org; 4 Budker Institute of Nuclear Physics, Siberian Branch of the Russian Academy of Sciences, 11, Acad. Lavrentieva Ave., 630090 Novosibirsk, Russia; darya.skorohod.88@gmail.com

**Keywords:** THz radiation, pyroelectric detector, polyvinylidene difluoride, lithium tantalite, tetra-aminodiphenyl, noise equivalent power, free electron laser

## Abstract

The development of efficient and reliable sensors operating at room temperature is essential to advance the application of terahertz (THz) science and technology. Pyroelectric THz detectors are among the best candidates, taking into account their variety, outstanding performance, ease of fabrication, and robustness. In this work, we compare the performance of six different detectors, based on either LaTiO_3_ crystal or different polymeric films, using monochromatic radiation of the Novosibirsk Free Electron Laser facility (NovoFEL) in the frequency range of 0.9–2.0 THz. The main characteristics, including noise equivalent power and frequency response, were determined for all of them. Possible reasons for the differences in the obtained characteristics are discussed on the basis of the main physicochemical characteristics and optical properties of the sensitive area. At least three detectors showed sufficient sensitivity to monitor the shape and duration of the THz macropulses utilizing only a small fraction of the THz radiation from the primary beam. This capability is crucial for accurate characterization of THz radiation during the main experiment at various specialized endstations at synchrotrons and free electron lasers. As an example of such characterization, the typical stability of the average NovoFEL radiation power at the beamline of the electron paramagnetic resonance endstation was investigated.

## 1. Introduction

Pyroelectric sensors made on the basis of various thin crystals or polymeric films play an essential role in modern technology, medicine, and scientific research [1,2,3,4,5,6]. Such detectors use permanently poled ferroelectric materials capable of producing an electric current when absorbing incident radiation. These materials, especially lithium tantalite (LiTaO_3_) [7] and triglycine sulfate [8], have been successfully used in the infrared (IR) and terahertz (THz) frequency ranges and are admirable candidates for broadband detectors operating at room temperature. In addition to inorganic or organic single crystals, various polymeric pyroelectric sensors are currently matured technological products. Polarized polymers such as polyvinylidene difluoride (PVDF) or 2,2′,4,4′-tetra-aminodiphenyl (TADP) demonstrate strong and stable piezoelectric and pyroelectric activities, giving rise to their abundant practical applications [9,10,11,12]. The attractiveness of polymers is determined by their low cost, flexibility, mechanical and chemical resistance, and the possibility to be deposited on various types of substrates. Recent advances in pyroelectric sensor technology, comprising both single crystal and polymeric materials, include: (i) pulsed-laser detectors based on composite materials [13]; (ii) biomedical system-on-a-chip [14]; (iii) integrated sensors [15,16]; (iv) detectors for THz time-domain spectroscopy and imaging [17,18,19,20]; (v) detectors for optoacoustic microscopy [21,22]; (vi) the use of the terajet effect and Fano response to improve performance of detectors [23,24]; (vii) 3D-printed detectors [25]; (viii) X-ray generators [26], etc.

Characterization of the pyroelectric transducers in the far-infrared and terahertz regions is usually performed by measuring the broadband response of the detector to blackbody radiation of a Fourier transform infrared spectrometer (FTIR) source [27,28]. The use of monochromatic radiation is usually limited to HeNe or CO_2_ lasers [29] with a few exceptions [30], since similar radiation sources in the THz region are not common [31,32]. There is also a lack of direct comparisons of different sensors made under identical or at least similar experimental conditions. Such comparisons are mainly accomplished in reviews [2]. In this paper, using the unique capabilities provided by the Novosibirsk Free Electron Laser (NovoFEL) facility, we characterized the performance of several different detectors at four wavenumbers: 66.7, 50.8, 41.7, and 28.6 cm^−1^. Given the rapid progress in terahertz science and technology, a detailed comparison of sufficiently cheap and widespread detectors is essential to wisely select a suitable transducer for any practical applications, including the development of specialized THz endstations at synchrotrons or free electron lasers. Six detectors of different kinds were used: two commercially available detectors and four home-made ones based on poled PVDF film coated with different electrodes. For all of them, noise equivalent power (NEP) was measured at the specified wavenumbers. Their frequency response and linearity were also determined. The optical properties of the PVDF film with different electrodes were characterized by FTIR spectroscopy, which allowed us to find correlations between them and the obtained NEP. Finally, we describe the typical values of NovoFEL radiation instabilities and a possible way to correct them by controlling the shape and duration of the THz macropulses during experiments using one of the investigated detectors as an example.

Section 2 of the article describes the radiation source, the layout of the detectors at the endstation, the preamplifier used for home-made detectors, and a comparison of the main physicochemical characteristics of the sensitive area of the detectors. Section 3 presents the obtained NEP values, frequency responses, linearity check, and two application examples of the detectors at the electron paramagnetic resonance endstation.

## 2. Materials and Methods

### 2.1. Radiation Source—Novosibirsk Free Electron Laser Facility

The performance of the pyroelectric detectors was investigated using monochromatic radiation from NovoFEL with four different wavenumbers: 66.7 cm^−1^ (150 μm; 2.0 THz); 50.8 cm^−1^ (197 μm; 1.5 THz); 41.7 cm^−1^ (240 μm; 1.25 THz); 28.6 cm^−1^ (350 μm; 0.85 THz). The radiation spectra are shown in Figures S1–S4 of the Supplementary Material. The NovoFEL facility includes three free electron lasers (FELs) operating in the terahertz, far-infrared, and mid-infrared spectral ranges [33,34]. Since its launch to users in 2004, terahertz FEL remains the most powerful source of coherent narrowband (δλ/λ=0.2–2%) radiation in the world in the 25–111 cm^−1^ (90–400 μm; 0.75–3.3 THz) frequency range. Its average power of radiation reaches 0.5 kW and its peak power is about 1 MW. The unique radiation parameters are provided using the energy recovery linear accelerator for electron acceleration and resonator-type FEL. All three FELs work in quasi-continuous mode with a light pulse frequency of ~5.6 MHz, determined by the length of the optical resonator. The duration of each pulse is about 100 ps. The radiation of each pulse is fully spatial and transverse coherent. The coherence between pulses strongly depends on the operation regime and can be observed in a sequence of up to 200 pulses [35,36]. Since various pyroelectric transducers are well described in the visible, near-infrared, mid-infrared, and far-infrared spectral ranges [27,29], only THz radiation in the 0.9 to 2 THz range was used in this work. The NovoFEL facility can also operate in the so-called macropulse lasing mode [37]. In this regime, the electron beam consists of macropulses of electron bunches, phased with the FEL optical resonator and separated by unsynchronized bunches. This makes it possible to switch between quasi-continuous mode and macropulse regime without overloading the accelerating system of the facility. The minimum duration of the macropulse is determined by the optical resonator and is usually a few microseconds. Given this limitation, there are no other practical constraints on the time profile of the THz macropulse, including its maximum duration, repetition rate, and duty cycle. Macropulse fronts at a certain wavenumber depend on the ratio of optical mode losses and the FEL gain for the macropulse rise time, and on the quality factor of the optical cavity for the macropulse decay time. A duty cycle of 2 was used in all measurements, unless otherwise specified. The detectors have been characterized and are currently being used at the THz beamline of the electron paramagnetic resonance (EPR) spectroscopy endstation, which allows the study of paramagnetic species in various media using continuous wave and time-resolved EPR techniques [38,39].

### 2.2. Characterization of the Detectors

Two classes of the detectors were used: (i) commercially available detectors combined with a preamplifier in a standardized “metal can” semiconductor package; (ii) home-made pyroelectric detectors based on bioriented poled PVDF film coated with different electrodes.

Commercial detectors under study are QS-IF5 (Gentec-EO, Quebec, QC, Canada) and MG-32 (Vostok, Novosibirsk, Russia). The QS-IF5 pyrodetector has a sensitive area of 5 mm and is based on a thin LiTaO_3_ crystal, which has a high pyroelectric coefficient of up to 480 μC·m^−2^·K^−1^ [40]. The sensing unit of the QS-IF5 has a metallic coating of unknown thickness, and it is exposed to radiation without a protective window. The MG-32 detector has a sensitive area of 1 mm made of a 1 µm thick polymer film of TADP. The sensing element of the MG-32 has a 30 nm thick aluminum coating applied by sputtering, has no protective window, and is directly attached to a polypropylene lens. The addition of the lens was a special modification made by the manufacturer. The materials, thicknesses, and sensitive areas listed are summarized in Table 1. Detectors based on PVDF films are described in Section 2.3.

**Figure 1:**
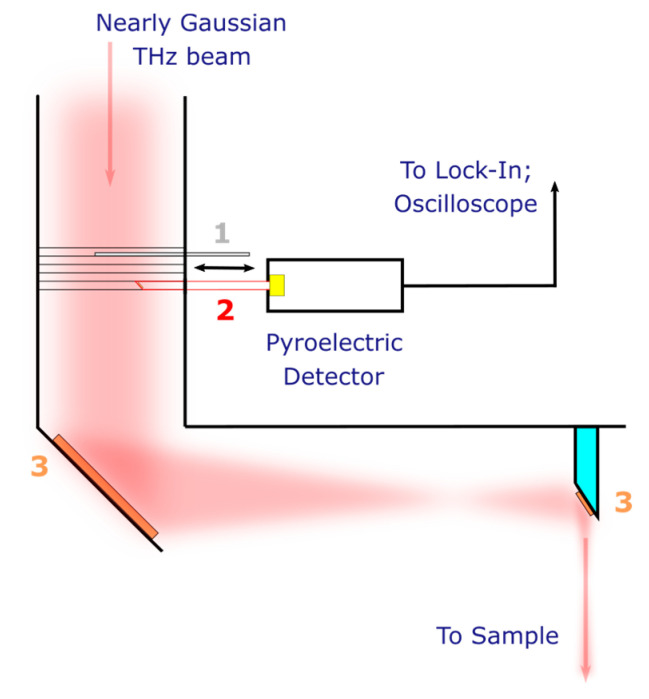
The layout of the pyroelectric detector at the THz beamline of the EPR spectroscopy endstation. Numbers show: 1—movable mechanical shutter; 2—movable copper tube with outer and inner diameters of 8 and 6 mm, respectively, and a copper mirror at one end; 3—off-axis parabolic mirrors. THz beam at the entrance of the optical system is nearly Gaussian [41].

Figure 1 shows the layout of the investigated detectors at the EPR spectroscopy endstation. The detector in an aluminum case is mounted on a movable hollow copper tube. At one end of the tube there is a copper mirror located at an angle of about 45° to the incident almost Gaussian beam of THz radiation [41]. The second end of the tube is 1–2 mm from the sensitive area for all the transducers except MG-32, for which the tube rests against the polypropylene lens. Placing the tube in front of the first focusing element of the optical system only slightly reduces the total power, at the same time allowing one to control the shape and duration of the NovoFEL macropulse during experiments (see Section 3.4). This also ensures that the sensitive area of the detector is uniformly irradiated, which is important when measuring noise equivalent power. Photographs of the detector and copper tube placed in the optical system of the EPR endstation are given in Figure S5 of the Supplementary Material.

**Table 1:** Comparison of the main characteristics of the investigated transducers.

Detector Name		QS-IF5	MG-32	ITO	Cu/Ni	Au	Ag-ink
Sensitive element	Material	LaTiO_3_	TADP ^a^	PVDF ^b^	PVDF ^b^	PVDF ^b^	PVDF ^b^
	Thickness (μm)	— ^c^	1	28	28	12	28
	Diameter (mm)	5	1 ^d^	4	4	4	4
Electrodes	Material	Me ^e^	Al ^f^	ITO ^f^	Cu/Ni ^f^	Au ^f^	Ag ^g^
	Thickness (nm)	— ^c^	30	2500 ^h^	70/10	100	10,000
Preamplifier feedback	Resistance (MΩ)	100	10^−3^	100	100	100	100
	Capacitance (μF)	—	0.22	—	—	—	—

a: polymer film of 2,2′,4,4′-tetra-aminodiphenyl; b: polymer film of polyvinylidene difluoride; c: exact numbers not provided by the manufacturer; d: polypropylene lens is directly connected to the sensitive area; e: exact metal is not provided by the manufacturer; f: sputtered metal electrodes; g: screen-printed electrodes; h: calculated based on ITO sheet resistance of 300 Ω/sq, as specified by the manufacturer, using a resistivity of 7.5 × 10^−4^ Ω·cm [42].

NEP (Section 3.1) was defined as the ratio of average radiation power to signal-to-noise ratio (SNR), measured in a 1 Hz output bandwidth [43]. Radiation power was determined by Ophir Juno (Ophir Optronics, Jerusalem, Israel) equipped with a calibrated 3A-P-THz sensor (Ophir Optronics, Jerusalem, Israel). To measure the power, the Ophir sensor was installed instead of the pyroelectric detector (see Figure 1). For simplicity, all power measurements were taken at duty cycle of 2 and then multiplied by the corresponding factor. Using a duty cycle of 1 (continuous radiation) gives the same results. The measured radiation power was recalculated to the sensitive area for all detectors except the MG-32, assuming a uniform distribution of radiation at the end of the copper tube. No correction was used for the MG-32 because it has a polypropylene lens directly attached to the sensitive area.

SNR was measured with a SR860 lock-in amplifier (Stanford Research System, Sunnyvale, CA, USA) using a time constant of 100 ms and a low-pass filter of 12 dB/oct that corresponds to f_−3dB_ of 1.02 Hz. The QS-IF5 and MG-32 detectors contain a hybrid preamplifier, so they were directly connected to lock-in by a BNC cable. The lock-in amplifier was synchronized with the NovoFEL electronic modulation system. The signal from PVDF-based detectors was passed through a home-made preamplifier (see Section 2.4) and then through SR240A 300 MHz preamplifier (Stanford Research System, Sunnyvale, CA, USA), which additionally amplifies the signal by a factor of approximately 5. To obtain the SNR, 400 points were measured with the mechanical shutter open (see Figure 1), after which the same procedure was repeated with the shutter closed. The former data were used to calculate the signal level as a mean value, and the latter data were used to calculate the noise level as a standard deviation. The received signal was phase-corrected via multiplication by the factor exp(iφ), where φ was chosen so that the entire signal was in one channel. Therefore, only the X channel was analyzed to obtain the SNR. The measurements were automated using the open-source software Atomize (https://github.com/Anatoly1010/Atomize accessed on 1 October 2023).

The frequency characteristics (Section 3.2) were determined at 66.7 cm^−1^ (150 μm; 2.0 THz) in the range of 10–5000 Hz using the NovoFEL electronic modulation system, changing the macropulse repetition rate, while maintaining a duty cycle equal to 2. The upper frequency limit is determined by the minimal reasonable macropulse length, which is 50–100 μs in the 0.9–2.0 THz range.

The linearity of the response of the studied detectors (Section 3.3) was verified at 41.7 cm^−1^ (240 μm; 1.25 THz) by comparing their response with the calibrated Ophir sensor. The maximum average radiation power was approximately 20 mW. It was further attenuated by placing several 1 mm thick polyethylene terephthalate films in front of the mechanical shutter (see Figure 1).

The time profiles of the THz macropulses were obtained using a 350 MHz Keysight DSOX3034T oscilloscope (Keysight Technologies, Santa Rosa, CA, USA) to which the output signal from the detectors was connected. The NEP was also calculated from the measured macropulse time profiles using half the sampling frequency as the noise equivalent bandwidth. The obtained numbers coincide with the NEPs measured by the lock-in amplifier within 10% and are not provided. The arbitrary wave generator of the same oscilloscope was used to trigger the electronic modulation system. The spectral power densities of the noise were obtained by a fast Fourier transform of the oscillograms, measured in the 1 or 5 s time windows.

FTIR transmittance and reflectance spectra of PVDF films with different coatings were measured using a Bruker Vertex 80v FTIR spectrometer (Bruker, Billerica, MA, USA) in the far-infrared range using a room temperature PE/DLaTGS D201 detector. The reflectance spectra were measured using A510/Q-T 11 combined transmission and specular reflection accessory.

### 2.3. PVDF-Based Detectors

Four different types of electrodes that cover the PVDF film on both sides were investigated, namely indium tin oxide (ITO), Cu/Ni, Au, and Ag. All of them were manufactured by PolyK (PolyK Technologies, Philipsburg, PA, USA) using either metal sputtering (ITO, Cu/Ni, Au) or screen printing (Ag). A comparison of the material and thickness of the electrodes, as well as the thickness of the PVDF film, is given in Table 1. Pyroelectric coefficient of PVDF is 30 μC·m^−2^·K^−1^, according to the manufacturer. Hereinafter, pyroelectric detectors based on PVDF films are named the same as electrodes, with the exception of Ag-coated film, where the name Ag-ink is used to emphasize a different method of manufacture. The sensitive area of the PVDF-based detectors had a diameter of 4 mm and was defined by a hole in the copper clad laminates, which also play the role of electric contacts (Section 2.4). The entire PVDF film placed between the contacts was 6 mm square. When a square piece of PVDF film was replaced with a similar piece, the measured NEP values varied within 5%, indicating good quality of film coating. A photograph of the films under study before their placement between the copper clad laminates is given in Figure S6 of the Supplementary Material.

**Figure 2:**
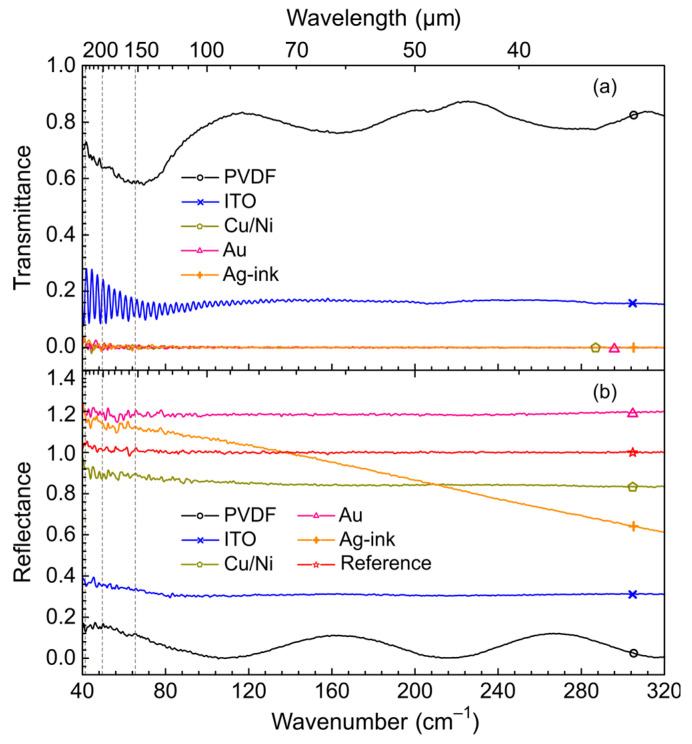
(**a**) FTIR transmittance spectra of the studied PVDF films with different electrodes, as well as of pure PVDF of 28 μm thickness in the far-infrared spectral range. Symbols and line colors show: black, “○”—PVDF; blue, “x”—ITO; dark green, “⬠”—Cu/Ni; pink, “△”—Au; orange, “+”—Ag-ink. The Cu/Ni, Au, and Ag-ink spectra are superimposed. (**b**) Same as (**a**) for reflectance. The red line marked with an asterisk “☆” shows the reference signal of the gold plate used. The vertical dashed lines show the wavenumbers at which the NEP values were determined.

The transmittance and reflectance of PVDF films with different coatings as well as pure PVDF were studied in the far-infrared spectral range using 3 cm squared pieces mounted in A510-H sample holder of the FTIR spectrometer. According to Figure 2a,b, pure PVDF film demonstrates the average transmittance of about 80% over the shown energy range. The average reflectance (neglecting interference) is about 10% mainly due to reflection at the boundary of media with different optical densities (ε_PVDF_ is about 13.0, according to the manufacturer). Thus, the possible absorption of radiation by the 28 μm thick PVDF film does not exceed 10%, and its optical properties are determined mainly by the coatings used. Sputtering the PVDF film with a semiconductive ITO material reduces transmittance to 20% uniformly over the entire energy range investigated, while reflectance increases to 30–35%. Based on the percentage of transmitted and reflected energy, one can assume that the rest of the radiation (~40%) is absorbed in the ITO layer. Films with sputtered (Cu/Ni, Au) or screen-printed (Ag-ink) metal coatings are impervious to incident radiation in the range of 40–320 cm^−1^. At the same time, the reflection of these films is close to the reflection of the gold plate used in this experiment as a reference. The reflection behavior of the Ag-ink film is probably determined by scattering of radiation on the particles forming the coating. Their size, apparently, is comparable with the wavelength of radiation in the short wavelength part of the spectrum (~30 μm), which gives rise to a monotonic decrease in the reflection level with wavelength. Considering the transmittance and reflectance levels of Cu/Ni, Au, and Ag-ink coated films, one would expect the absence of significant absorption by these films. Nevertheless, the reflectance of Cu/Ni is still lower than that of Au, i.e., Cu/Ni probably absorbs more incident radiation than Au that correlates with the obtained NEP (see Section 3.1).

### 2.4. Preamplifier

Commercially available pyroelectric detectors are usually combined with a preamplifier in a small “metal can” semiconductor package. In order to compare their performance with cheap PVDF-based detectors with different electrodes, a preamplifier was made for the latter. It was assembled according to the current-to-voltage converter circuit. An operational amplifier (OA) LTC6268 with extremely low input bias current (on the order of a few fA) and low input capacitance was used as an amplifier. The signal amplitude recorded by the PVDF-based sensors is in the order of 1–5 mV at 100 to 5000 MΩ, i.e., the current produced by the sensor is in the order of 1–10 pA. Therefore, the influence of the input currents of the OA on the resulting signal can be neglected. An additional amplifier was used to create a virtual ground. The preamplifier was powered by three AA batteries and enclosed in an aluminum case to reduce the influence of external interference. The complete circuits are provided in Figure S7 of the Supplementary Material. Electric contacts with PVDF electrodes are carried through two copper clad laminates with tinned holes. A PVDF film is placed between them and then they are clamped with an aluminum cover. Detailed photographs of the assembled preamplifier and contact plates are shown in Figures S5 and S6 of the Supplementary Material. No additional heat sink except for copper clad laminates was used.

## 3. Results and Discussion

### 3.1. Noise Equivalent Power

Using monochromatic radiation of the NovoFEL at four different wavenumbers, the corresponding NEPs at 500 Hz (QS-IF5, ITO, Cu/Ni, Au), 250 Hz (MG-32), or 20 Hz (Ag-ink) were determined for all the pyroelectric transducers studied. The exact frequency was chosen based on the response of the detectors (see Section 3.2). The data obtained for all considered detectors are summarized in Table 2.

The experimental results presented in Table 2 can be summarized in six main theses: (i) NEPs of commercial detectors are at least two orders of magnitude better than those of PVDF-based ones, which is probably determined by the pyroelectric coefficient of the sensitive material; (ii) the MG-32 detector based on TADP polymer film shows the best sensitivity; (iii) there is no significant spectral dependence of the NEP in the investigated energy range; (iv) among PVDF-based detectors, the lowest NEP shows ITO, which is a consequence of higher radiation absorption (see Section 2.3); (v) the NEP of other PVDF-based detectors also correlates with the optical properties of their coatings; (vi) the results show good reproducibility in independently performed experiments.

In more detail, the two commercial detectors studied have NEP in the order of dozens of nW·Hz^−1/2^ in the 0.9–2.0 THz range, while the home-made analogues based on PVDF films have characteristics at least two orders of magnitude worse. The apparent difference in NEP appears to be caused by the difference in the pyroelectric coefficient that is 480 and 30 μC·m^−2^·K^−1^ for LaTiO_3_ (QS-IF5) and PVDF, respectively. Nevertheless, given the low cost and ubiquity of PVDF polymeric film, as well as the possibility to use the unique shape of the sensitive area and contact assembly of the electrodes, such detectors can be useful in certain circumstances, despite the higher NEP. For instance, they can be placed inside the MW cavity or waveguide of an EPR spectrometer [44,45].

For the QS-IF5 and MG-32 detectors, the NEP values are comparable to those for Schottky barrier diodes. To date, the latter detectors are probably the best known uncooled transducers in the THz frequency range, achieving NEP in the order of 10^−12^, 10^−10^, and 10^−9^ W·Hz^−1/2^ at 0.1, 0.9, and 2.52 THz, respectively [46,47,48,49]. Some metal bolometers and field-effect transistor-based terahertz detectors can also achieve NEP  in the order of  (2–5)⋅10^−11^ W·Hz^−1/2^ in the THz frequency range [50,51,52,53], while Golay cells show almost the same NEP as we obtained for the best pyroelectric transducers.

**Figure 3:**
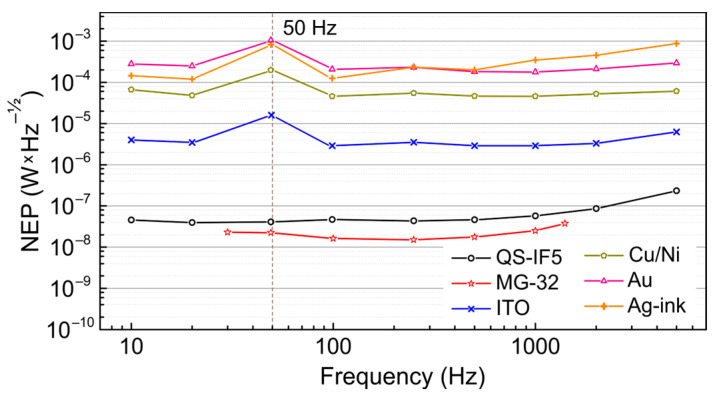
Frequency dependence of NEP measured at 28.6 cm^−1^ (350 μm; 0.85 THz). Symbols and line colors show: black, “○”—QS-IF5; red, “☆”—MG-32; blue, “x”—ITO; dark green, “⬠”—Cu/Ni; pink, “△”—Au; orange, “+”—Ag-ink. The frequency of 50 Hz is indicated by a dashed vertical line. The upper frequency of 5 kHz used is determined by the minimal reasonable THz macropulse length. Please note that the values were not measured at frequencies that are multiples of 50 Hz, but very close to them, i.e. not at 50 Hz, but at 49 Hz, to reduce line interference, but to keep the trend.

According to Figure 3, the frequency dependence of NEP measured at 28.6 cm^−1^ (350 μm; 0.85 THz) shows the same trend as the frequency response of the detectors (see Section 3.2). This means that there is no significant change in detector noise in the investigated frequency range. The only exception is the frequencies near 50 Hz for all PVDF-based detectors, where the NEP increased about fourfold due to an increase in detector noise. The same is observed in the noise power spectral densities that are shown in Figures S8–S13 of the Supplementary Material. This is at least partly a consequence of the use of the additional SR240A preamplifier. It was used to increase the noise level of these detectors higher than the intrinsic noise of the lock-in amplifier. The SR240A preamplifier was powered from the mains, not from the internal battery, and its noise power spectral density is shown in Figure S14.

The visible transparency of the ITO coated film allows a visually inspected additional manual coating of the electrode. Graphite was used as an example because, according to the literature, it has decent absorption in the THz range [14,28,54,55,56]. Graphite was applied to the electrode of the ITO detector with a pencil, and its NEP was measured again. No significant difference in the NEP value was obtained that probably indicates insufficient thickness of the graphite layer for a meaningful increase in absorption.

### 3.2. Frequency Response

**Figure 4:**
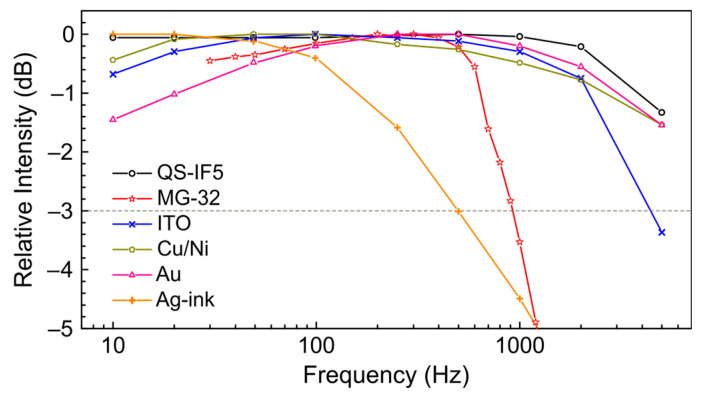
Frequency response of the detectors measured at 66.7 cm^−1^ (150 μm; 2.0 THz). Symbols and line colors show: black, “○”—QS-IF5; red, “☆”—MG-32; blue, “x”—ITO; dark green, “⬠”—Cu/Ni; pink, “△”—Au; orange, “+”—Ag-ink. The dashed horizontal line indicates the −3 dB level. The upper frequency of 5 kHz used is determined by the minimal reasonable THz macropulse length.

Figure 4 shows the frequency response of the transducers measured at 66.7 cm^−1^ (150 μm; 2.0 THz) in the range of 10–5000 Hz. Relevant information about the resistance and capacitance in the preamplifier feedback is given in Table 1. Let us firstly discuss the high frequency part of the response. The MG-32 shows a significant signal drop above 700 Hz that is caused by the RC of the preamplifier feedback. In contrast, the ITO and Ag-ink detector signal fall off is controlled by the intrinsic temporal response of the films and their coatings. The behavior of the other detectors, namely QS-IF5, Cu/Ni, Au, is somewhat more confusing, since their frequency characteristics are comparable to both RC and the rise and decay times of the NovoFEL radiation caused by its optical resonator. In the case of reduced resistance in the preamplifier feedback, the QS-IF5 allows the detection of a fine structure of the NovoFEL macropulses, as shown in Figure S15 of the Supplementary Material. In contrast, the Cu/Ni and Au detectors demonstrate almost the same behavior as was detected with 100 MΩ resistance. This means that their frequency response is governed by their intrinsic temporal response, while for the QS-IF5 it is limited by the RC of the preamplifier feedback. The temporal response of the detectors, if has been observed, correlates with the total thickness of the sensitive area and electrodes. The thinner the film and the electrodes, see Table 1, the faster the temporal response and the wider the detector bandwidth. The time profiles of the NovoFEL macropulses obtained by all detectors are given in Figures S15–S17 of the Supplementary Material.

As for the low frequency part of the response, for the MG-32, ITO, Au, and Cu/Ni detectors there is a noticeable drop in the measured signal at frequencies below 20–100 Hz. The most pronounced effect is observed for the Au detector that, according to Table 1, has the thinnest sensitive area among PVDF-based detectors. This means that the effect may be related to the macroscopic thermal relaxation of the sensitive area of the detectors, which obviously depends on the overall thickness. The behavior of the MG-32 cannot be directly compared because it has a different design and a different heat sink as a consequence. Nevertheless, the thickness of the sensitive area of the MG-32 is only 1 µm.

### 3.3. Linearity of the Detector Response

**Figure 5:**
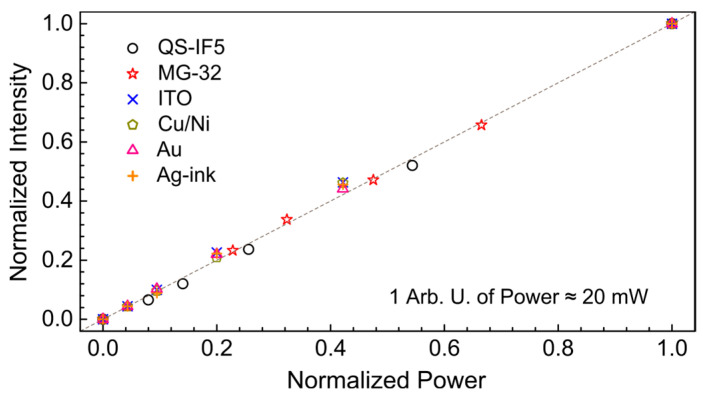
Linearity of the detector responses measured at 66.7 cm^−1^ (150 μm; 2.0 THz) using a calibrated 3A-P-THz sensor. Symbols and line colors show: black, “○”—QS-IF5; red, “☆”—MG-32; blue, “x”—ITO; dark green, “⬠”—Cu/Ni; pink, “△”—Au; orange, “+”—Ag-ink. The dashed line is a guide for eyes. One arbitrary unit of power corresponds to 20 mW. One arbitrary unit of intensity depends on the detector used and varied from a few mV to several V.

According to Figure 5, all investigated detectors exhibit linear behavior up to at least 20 mW of applied averaged power. Higher power was not used since it is rarely achieved in experiments, taking into account the layout of the detectors at the EPR endstation, (see Figure 1). Nevertheless, all the detectors are resistant to short-term use of average power in the order of 100–500 mW in the range of 0.9–2.0 THz. As a test experiment, high-power focused radiation was applied to the PVDF-based ITO detector. An average power of about 2–3 W was able to burn a hole in the sensing area that, however, did not lead to the complete destruction of the detector, since the electrodes were not short-circuited.

### 3.4. Application at the EPR Endstation

**Figure 6:**
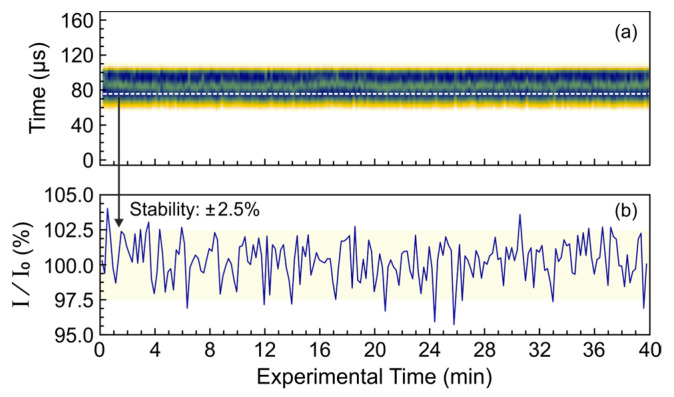
THz macropulses with a duration of 60 μs, a wavenumber of 76.9 cm^−1^ (130 μm; 2.3 THz), and a repetition rate of 2.3 Hz measured with the QS-IF5 detector in parallel with the experiment. (**a**) Two-dimensional plot showing the shape of the macropulses and their horizontal jitter; (**b**) the time profile of the signal stability in the middle of the macropulses (shown in (**a**) by the white dashed line).

The regular operating mode of the NovoFEL does not provide for day-and-night operation. It switches on and off every day, resulting in a long period of thermal stabilization. During this period, there can be significant instability in the parameters of the electronic systems, primarily in the phases of the radio frequency resonators that accelerate the electron beam. The cathode emission also changes over the operating time. In addition, despite the use of active thermal stabilization circuits, there is a time-dependent heating of the mirrors and the walls of the optical resonator that leads to a change in the synchronization of the light and electron bunches. These effects affect the output power of radiation, so it is necessary to control the shape and duration of the THz macropulses during experiments. Such a possibility is realized at the EPR endstation, using the optical system described in Section 2.2 and one of the pyroelectric detectors utilized in this work. Figure 6 shows a typical example of the registered THz macropulses during an experiment lasting 40 min.

According to Figure 6b, the NovoFEL is capable of generating stable macropulses during a fairly long 40 min experiment. The maximum variation in THz power during the experiment is ±2.5%, which is comparable with the detector noise. There is also no significant horizontal jitter (see Figure 6a) that is even more important for experimentalists because it cannot be easily corrected, but can significantly distort the results of time-resolved experiments. Because of the high duty cycle (500–10,000) typically used at the EPR endstation, the entire time profiles of the macropulses are recorded with the oscilloscope instead of using lock-in detection.

**Figure 7:**
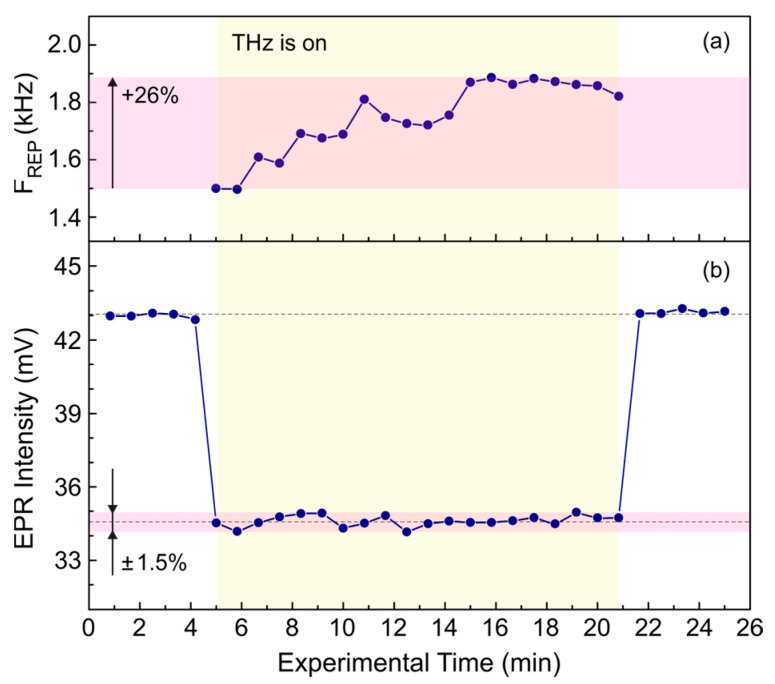
(**a**) Adjustment of the repetition rate of the THz macropulses used in the experiment. Macropulses with a duration of 100 μs and a wavenumber of 1118 cm^−1^ (8.95 μm; 33.5 THz) were measured by the QS-IF5 detector in parallel with the experiment. The repetition rate was varied to have the same integral intensity of the macropulse. (**b**) Stability of the EPR signal resulting from the influence of the THz macropulses after adjustment of the repetition rate.

The stability of the average THz radiation power over long periods of time is strongly dependent on the wavenumber of the THz radiation. At a wavenumber of about 77 cm^−1^ (130 μm; 2.3 THz), the stability is sufficient for typical experiments (see Figure 6), whereas, for example, at 1100 cm^−1^ (9 μm; 33.3 THz), the parameters are worse. Therefore, when working with mid-infrared laser radiation, stabilization of the radiation power is required. A practical example of an EPR experiment performed at 1118 cm^−1^ (8.95 μm; 33.5 THz) with poor stability of the radiation power is shown in Figure 7. In the experiment, irradiation of the system under study by THz radiation leads to a decrease in the EPR signal due to heating of the sample, which does not depend on the irradiation time. To stabilize the averaged THz power applied to the sample over the entire long-term experiment, the repetition rate of the THz macropulses was adjusted using the integral intensity of the macropulse measured by the QS-IF5 detector, as shown in Figure 7a. The change in the repetition rate was up to 26% that is large enough to substantially distort the experimental results. The correction applied kept the resulting EPR signal stable within a range of approximately ±1.5%, improving the long-term stability of the average THz power by more than an order of magnitude.

To summarize, the QS-IF5, MG-32, and to some extent ITO detectors are sensitive enough to monitor the NovoFEL radiation parameters concurrently with the experiments conducted at the endstation. This feature enhances the quality of the data acquired and helps prevent incorrect interpretations, caused by the presence of inevitable factors affecting the radiation power.

## 4. Conclusions

The results indicate differences in the performance among different types of pyroelectric detectors based on LaTiO_3_ crystal, TADP or PVDF polymeric films when exposed to pulsed THz radiation in the range of 0.9–2.0 THz. The noise equivalent power, measured using monochromatic radiation of the NovoFEL at 66.7, 50.8, 41.7, and 28.6 cm^−1^, is on the order of tens of nW·Hz^−1/2^ for commercially available detectors and at least two orders of magnitude higher for home-made PVDF-based detectors. The MG-32 detector based on the TADP polymer shows the best sensitivity, which is probably caused by the highest pyroelectric coefficient and the better quality of the embedded preamplifier. Among the four PVDF-based detectors studied, the film, whose electrodes are coated with ITO, has the lowest NEP of several μW·Hz^−1/2^, which is determined by the optical properties of the coating. Despite their limited performance, PVDF-based detectors can be useful for monitoring radiation at synchrotron and free electron laser IR and THz endstations, given their low cost, flexibility, mechanical and chemical resistance, and simple possibility to use a large sensitive area. All the detectors show no significant spectral dependence of the NEP in the investigated frequency range of 0.9–2.0 THz. The frequency response of the MG-32 and QS-IF5 detectors were limited by the RC of the preamplifier feedback. In the case of PVDF-based detectors, the response is controlled by the intrinsic temporal response of the sensitive area. Typical values are hundreds of microseconds for the Ag-ink detector and tens of microseconds for the Cu/Ni and Au detectors, while the ITO shows intermediate numbers. In the case of reduced RC, the QS-IF5 is able to detect the fine structure of the NovoFEL THz macropulse. It means that the intrinsic temporal response of the QS-IF5 is below a microsecond. The pyroelectric transducers studied exhibit linear behavior up to at least 20 mW of applied averaged power and are tolerant to short-term exposure to average power on the order of 100–500 mW in the 0.9–2.0 THz range.

The stability of NovoFEL radiation power is affected by instabilities of several parameters, mainly related to thermal drift of various elements. This makes tracking the shape and duration of the THz macropulses during experiments highly desirable. The QS-IF5, MG-32, and, to some extent, ITO detectors have sufficient sensitivity to monitor the NovoFEL radiation in parallel with the experiment at the endstation. This was illustrated by two practical examples. They showed that in an experiment lasting 40 min the typical stability of the radiation power is on the order of ±2.5%, and horizontal jitter is practically absent. If the average power of THz radiation applied to the sample is considered, the influence of power variation can be further reduced by adjusting the repetition rate of the THz macropulses.

## Figures and Tables

**Table 2:** NEP of the pyroelectric detectors measured using monochromatic laser radiation of the NovoFEL with different wavenumbers as indicated in the table.

Detector	NEP ^a^ (μW·Hz^−1/2^)				
	Wavenumber (cm^−1^); Wavelength (μm); Frequency (THz)				
	66.7; 150; 2.0	66.7; 150; 2.0 ^b^	50.8; 197; 1.5	41.7; 240; 1.25	28.6; 350; 0.85
QS-IF5 ^c^	2.2 × 10^−2^	1.8 × 10^−2^	2.8 × 10^−2^	2.9 × 10^−2^	4.7 × 10^−2^
MG-32 ^d,e^	1.3 × 10^−2^	1.6 × 10^−2^	1.5 × 10^−2^	1.0 × 10^−2^	1.3 × 10^−2^
ITO ^c^	2.3	2.5	2.2	3.1	2.8
Cu/Ni ^c^	41.8	33.8	23.0	37.3	50.3
Au ^c^	53.3	59.6	48.0	88.3	109.5
Ag-ink ^f^	28.9	26.7	23.7	37.2	144.3

a: specific detectivity D*, which is another valuable characteristic of the detector, can be calculated from the inverse NEP multiplied by a square root of the known sensitive area of the detector (see Table 1); b: measurement at 66.7 cm^−1^ (150 μm; 2.0 THz) was repeated twice on different days to check reproducibility; c: measured at 500 Hz; d: measured at 250 Hz; e: because of the polypropylene lens, the value was highly dependent on the alignment of the optical system and probably overestimated; f: measured at 20 Hz.

## Data Availability

The raw experimental data are available from the corresponding authors upon reasonable request.

## Supplementary Materials

The following supporting information can be downloaded at: https://www.mdpi.com/article/10.3390/polym15204124/s1, Figure S1: Radiation spectrum used for the performance study of the detectors at 66.7 cm^−1^ (150 μm; 2.0 THz); Figure S2: Radiation spectrum used for the performance study of the detectors at 50.8 cm^−1^ (197 μm; 1.5 THz); Figure S3: Radiation spectrum used for the performance study of the detectors at 41.7 cm^−1^ (240 μm; 1.25 THz); Figure S4: Radiation spectrum used for the performance study of the detectors at 28.6 cm^−1^ (350 μm; 0.85 THz); Figure S5: Photograph of the arrangement of the assembled preamplifier in the optical system and photograph of the front panel of the preamplifier; Figure S6: Photographs of 6 mm square pieces of PVDF film covered on both sides by four different types of electrodes and photograph of the contact plates on the two copper clad laminates; Figure S7: Circuit diagram of the preamplifier used to create a virtual ground and circuit diagram of the signal preamplifier; Figure S8: Noise power spectral density of the QS-IF5 detector in 7–400 Hz and 10–2000 Hz frequency ranges; Figure S9: Noise power spectral density of the MG-32 detector in 7–400 Hz and 10–2000 Hz frequency ranges; Figure S10: Noise power spectral density of the ITO detector in 7–400 Hz and 10–2000 Hz frequency ranges; Figure S11: Noise power spectral density of the Cu/Ni detector in 7–400 Hz and 10–2000 Hz frequency ranges; Figure S12: Noise power spectral density of the Au detector in 7–400 Hz and 10–2000 Hz frequency ranges; Figure S13: Noise power spectral density of the Ag-ink detector in 7–400 Hz and 10–2000 Hz frequency ranges; Figure S14: Noise power spectral density of the SR240A preamplifier with a 50 Ω load at the input instead of the signal in 7–400 Hz and 10–2000 Hz frequency ranges; Figure S15: Time profiles of NovoFEL the macropulses of 350 μs duration obtained at 66.7 cm^−1^ (150 μm; 2.0 THz) using the QS IF5 pyroelectric detector with two different resistances in the preamplifier feedback: 200 kΩ and 100 MΩ; Figure S16: Time profiles of the NovoFEL macropulses of 5 ms duration obtained at 66.7 cm^−1^ (150 μm; 2.0 THz) using the MG-32 pyroelectric detector; Figure S17: Time profiles of the NovoFEL macropulses of 1 ms duration obtained at 66.7 cm^−1^ (150 μm; 2.0 THz) using different PVDF based pyroelectric detectors.
