# Supplementary material for: Comparative Study of Single Crystal and Polymeric Pyroelectric Detectors in the 0.9–2.0 THz Range Using Monochromatic Laser Radiation of the NovoFEL
Source: Polymers (Basel). 2023 Oct 18;15(20):4124. doi: 10.3390/polym15204124 (PMC10610270; doi:10.3390/polym15204124)
Supplement: Supplementary file 1 [file polymers-15-04124-s001.zip › polymers-2580409-supplementary.pdf]

Supporting Information

Comparative Study of Single Crystal and Polymeric Pyroelectric Detectors in the 0.9–2.0 THz Range Using Monochromatic Laser Radiation of the NovoFEL

Anatoly R. Melnikov [1,2,*], Evgeny V. Kalneus [2,3], Yaroslav V. Getmanov [2,4], Darya A. Shevchenko [4], Vasily V. Gerasimov [2,4], Oleg A. Anisimov [3], Matvey V. Fedin [1,2] and Sergey L. Veber [1,2,*]

[1] International Tomography Center of the Siberian Branch of the Russian Academy of Sciences, 3a, Institutskaya Str., Novosibirsk 630090, Russian Federation; anatoly.melnikov@tomo.nsc.ru (A.R.M.); mfedin@tomo.nsc.ru (M.V.F.); sergey.veber@tomo.nsc.ru (S.L.V.)

[2] Novosibirsk State University, 1, Pirogova Str., Novosibirsk, 630090, Russian Federation

[3] Voevodsky Institute of Chemical Kinetics and Combustion of the Siberian Branch of the Russian Academy of Sciences, 3, Institutskaya Str., Novosibirsk 630090, Russian Federation; kalneus@kinetics.nsc.ru (E.V.K.); anisimov@academ.org (O.A.A.)

[4] Budker Institute of Nuclear Physics of the Siberian Branch of the Russian Academy of Sciences, 11, Acad. Lavrentieva Ave., Novosibirsk 630090, Russian Federation; y_getmanov@mail.ru (Y.V.G.); darya.skorohod.88@gmail.com (D.A.S.); v.v.gerasimov3@gmail.com (V.V.G.)

* Correspondence: anatoly.melnikov@tomo.nsc.ru (A.R.M.); sergey.veber@tomo.nsc.ru (S.L.V.)

Contents

1. Radiation Spectra Used to Study Detector Characteristics S2
2. Photograph of the Preamplifier and PVDF Films Used S4
3. Preamplifier Circuit Diagrams S5
4. Noise Power Spectral Densities S6
5. Time Profiles of the NovoFEL Macropulses Obtained with Different Detectors S9

1. Radiation Spectra Used to Study Detector Characteristics

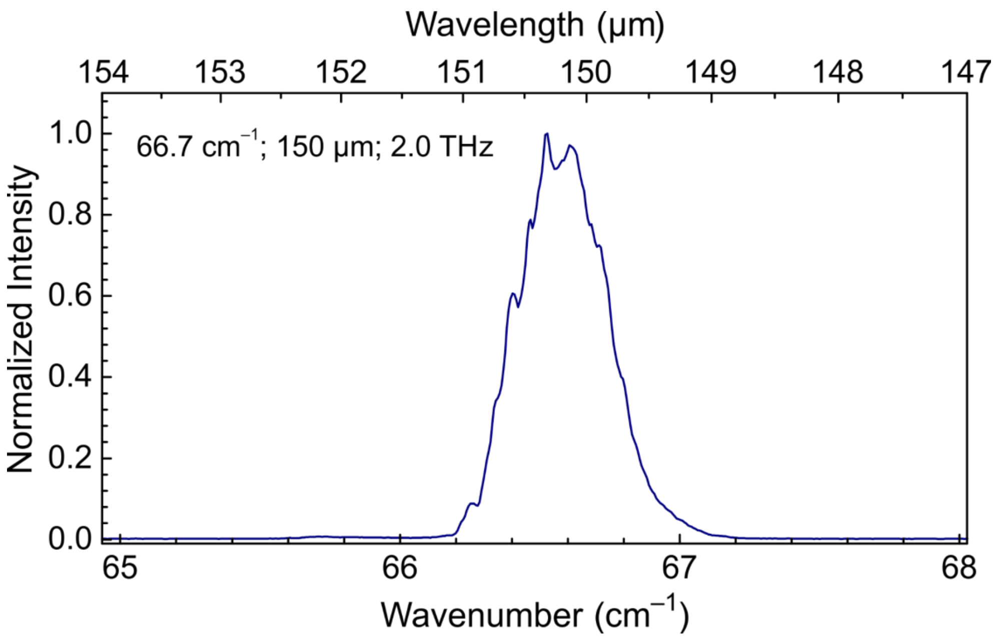

Figure S1. Radiation spectrum used for the performance study of the detectors at 66.7 cm^{-1} (150 µm; 2.0 THz).

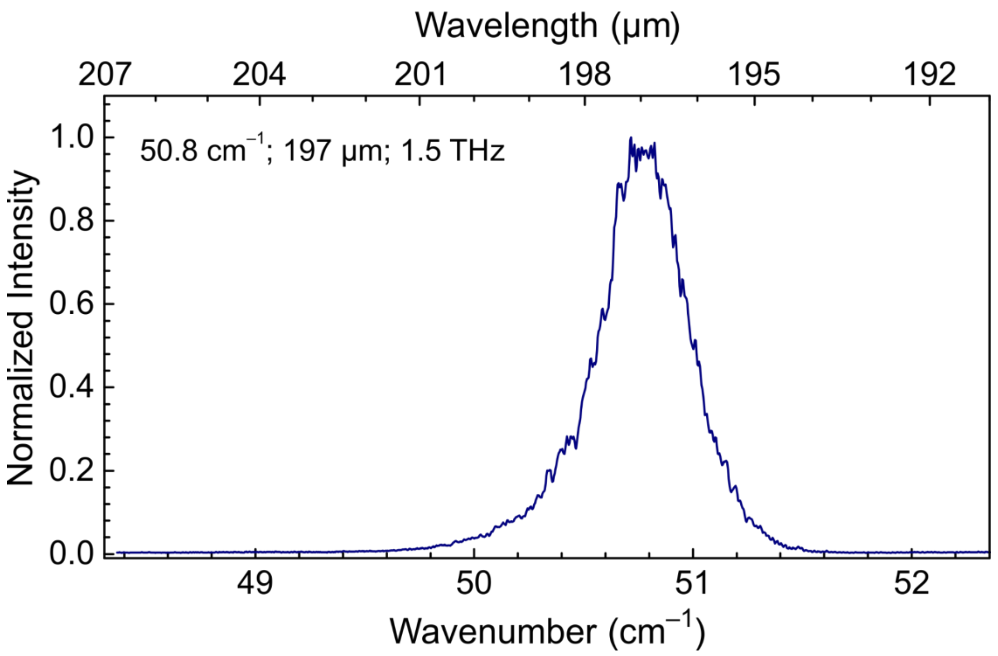

Figure S2. Radiation spectrum used for the performance study of the detectors at 50.8 cm^{-1} (197 µm; 1.5 THz).

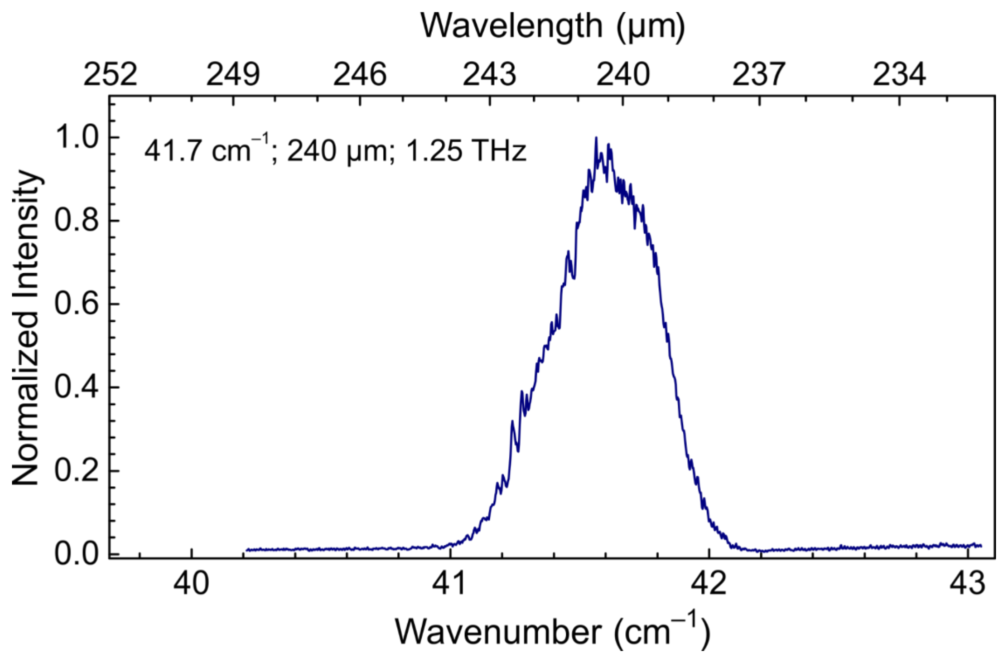

Figure S3. Radiation spectrum used for the performance study of the detectors at 41.7 cm^{-1} (240 μm; 1.25 THz).

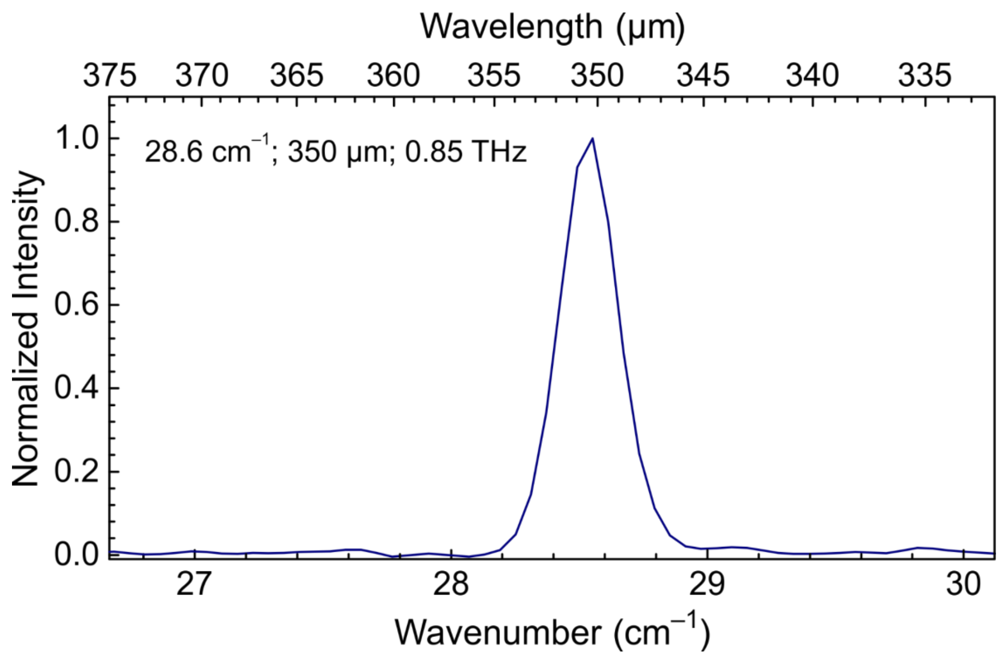

Figure S4. Radiation spectrum used for the performance study of the detectors at 28.6 cm^{-1} (350 μm; 0.85 THz).

2. Photograph of the Preamplifier and PVDF Films Used

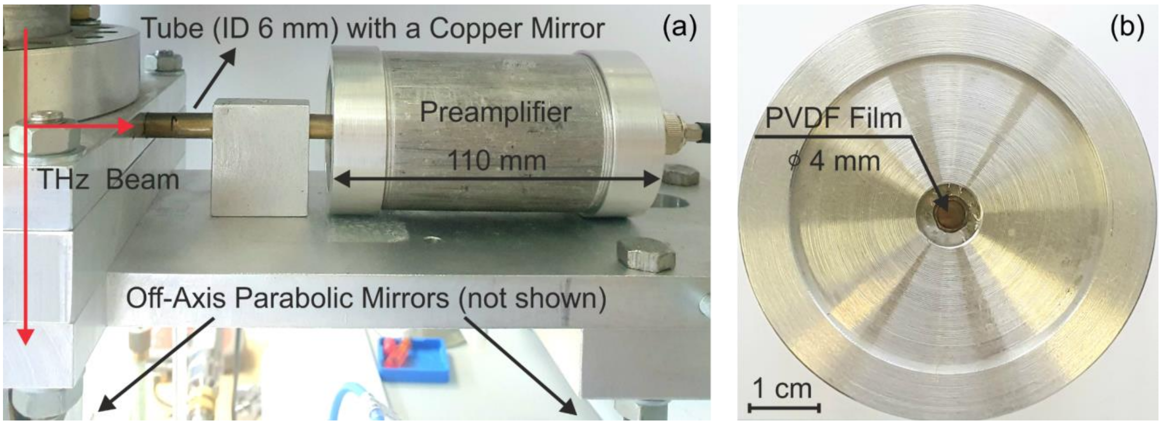

Figure S5. (a) Photograph of the arrangement of the assembled preamplifier in the optical system of the electron paramagnetic resonance endstation. (b) Photograph of the front panel of the preamplifier. One can see the sensitive area with a diameter of 4 mm made of PVDF film, coated, in this case, with Au.

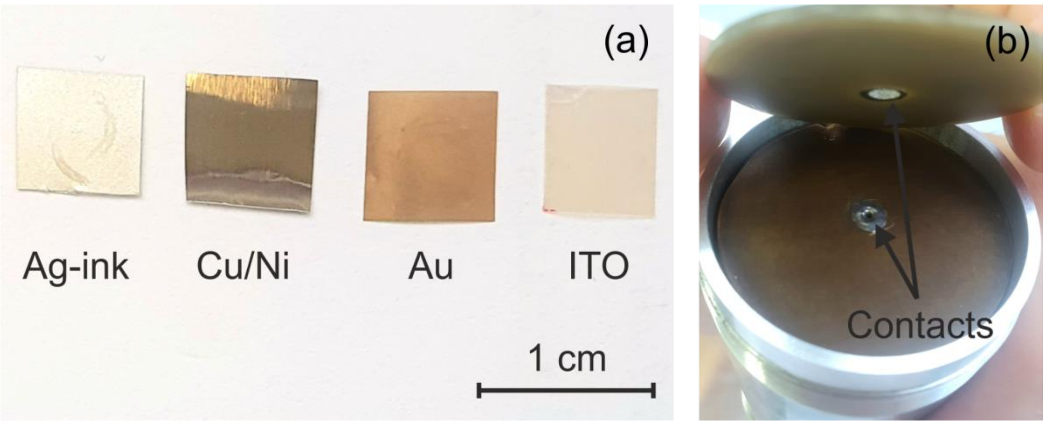

Figure S6. (a) Photographs of 6 mm square pieces of PVDF film covered on both sides by four different types of electrodes: indium tin oxide (ITO), Cu/Ni, Au, and Ag. The electrodes were made either by metal sputtering (ITO, Cu/Ni, Au) or screen printing (Ag). (b) Photograph of the contact plates on the two copper clad laminates.

3. Preamplifier Circuit Diagrams

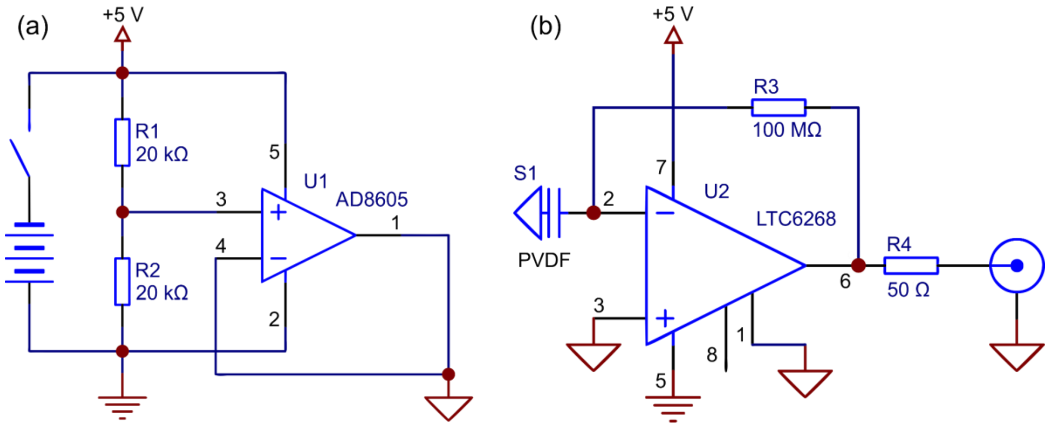

Figure S7. (a) Circuit diagram of the preamplifier used to create a virtual ground. (b) Circuit diagram of the signal preamplifier based on the current-to-voltage converter circuit.

4. Noise Power Spectral Densities

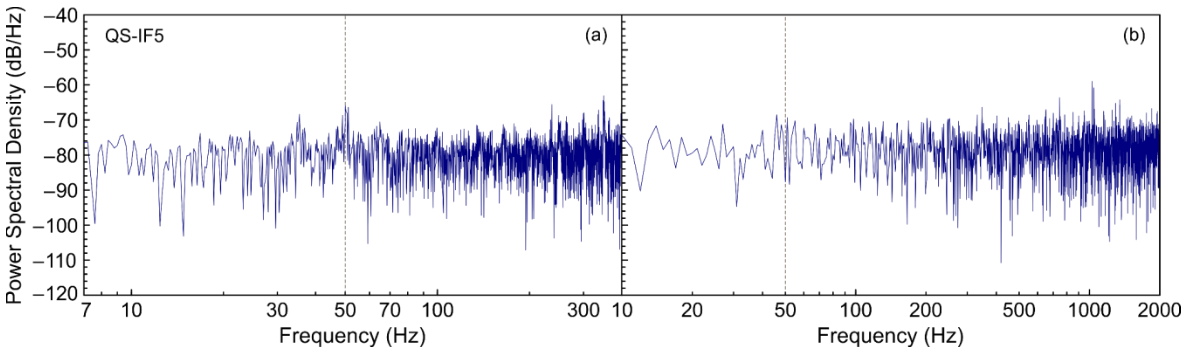

Figure S8. Noise power spectral density of the QS-IF5 detector in two frequency ranges: (a) 7–400 Hz in 0.2 Hz steps and (b) 10-2000 Hz in 1 Hz steps, obtained by fast Fourier transform of an oscillogram measured in time windows of 1 or 5 s, respectively.

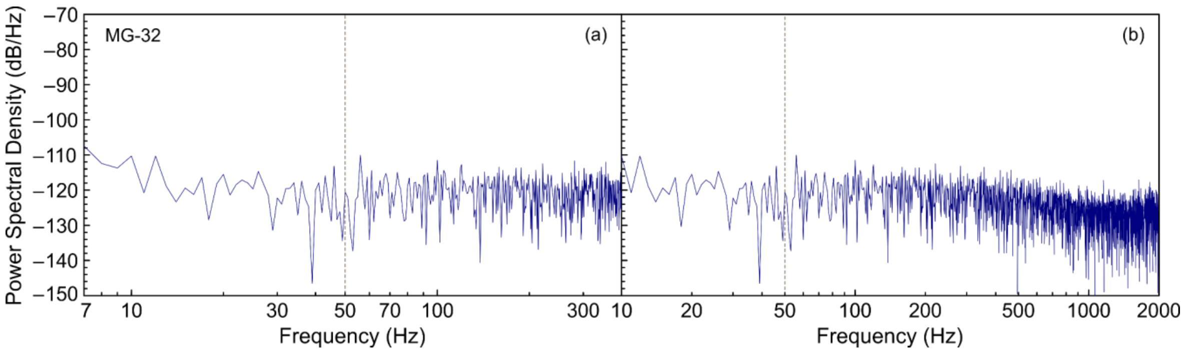

Figure S9. Noise power spectral density of the MG-32 detector in two frequency ranges: (a) 7–400 Hz in 0.2 Hz steps and (b) 10-2000 Hz in 1 Hz steps, obtained by fast Fourier transform of an oscillogram measured in time windows of 1 or 5 s, respectively.

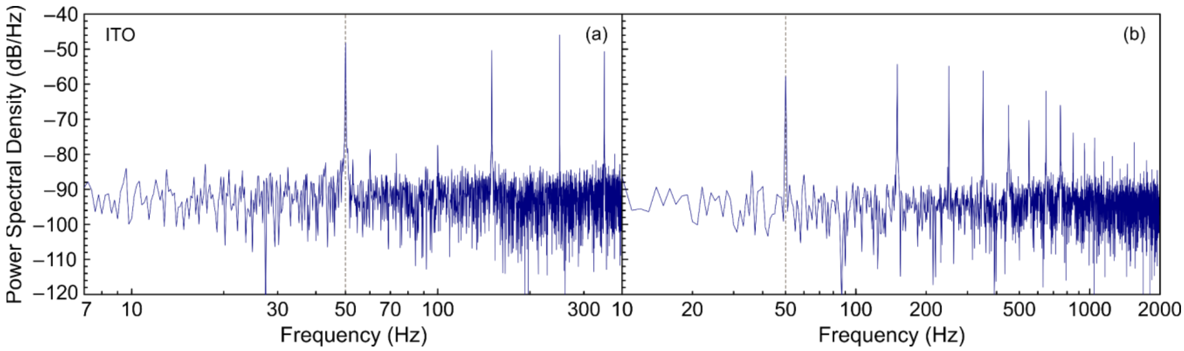

Figure S10. Noise power spectral density of the ITO detector in two frequency ranges: (a) 7–400 Hz in 0.2 Hz steps and (b) 10-2000 Hz in 1 Hz steps, obtained by fast Fourier transform of an oscillogram measured in time windows of 1 or 5 s, respectively.

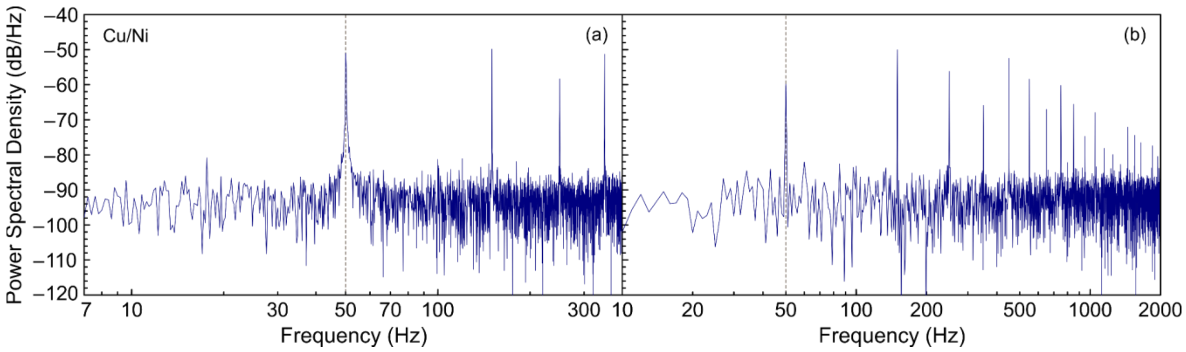

Figure S11. Noise power spectral density of the Cu/Ni detector in two frequency ranges: (a) 7–400 Hz in 0.2 Hz steps and (b) 10-2000 Hz in 1 Hz steps, obtained by fast Fourier transform of an oscillogram measured in time windows of 1 or 5 s, respectively.

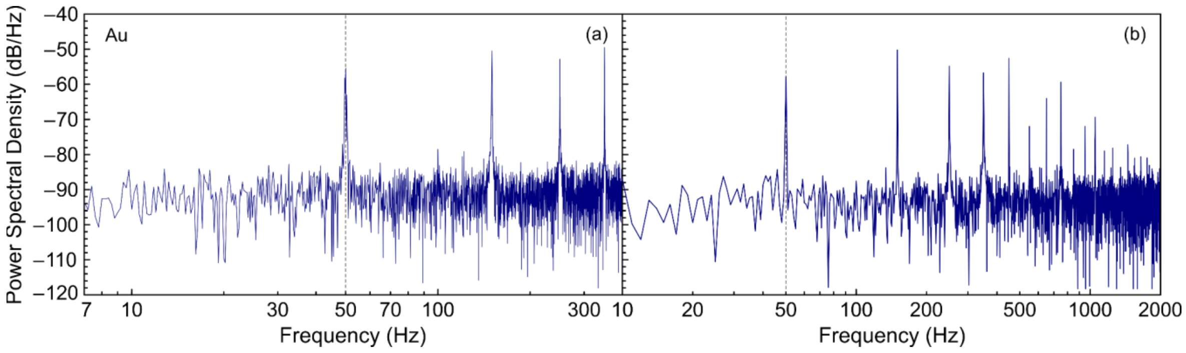

Figure S12. Noise power spectral density of the Au detector in two frequency ranges: (a) 7–400 Hz in 0.2 Hz steps and (b) 10-2000 Hz in 1 Hz steps, obtained by fast Fourier transform of an oscillogram measured in time windows of 1 or 5 s, respectively.

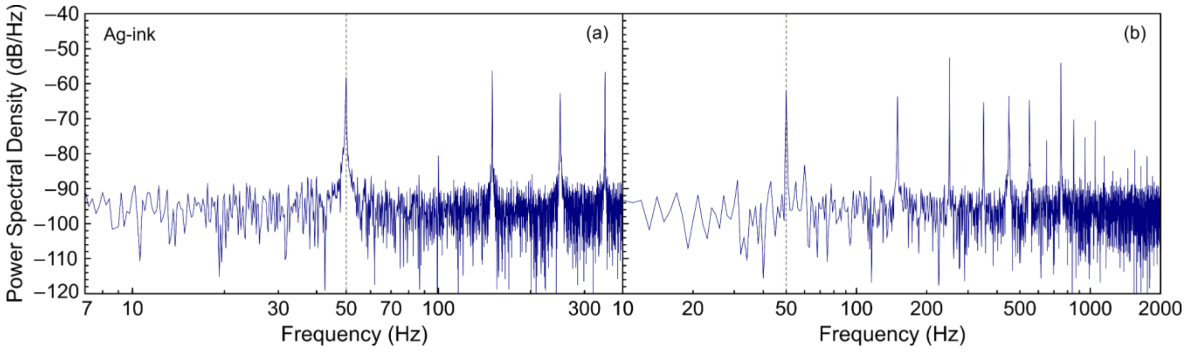

Figure S13. Noise power spectral density of the Ag-ink detector in two frequency ranges: (a) 7–400 Hz in 0.2 Hz steps and (b) 10-2000 Hz in 1 Hz steps, obtained by fast Fourier transform of an oscillogram measured in time windows of 1 or 5 s, respectively.

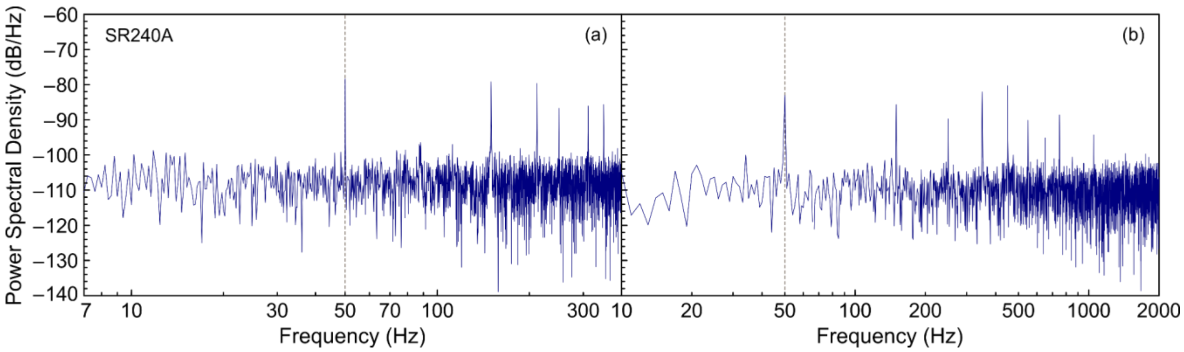

Figure S14. Noise power spectral density of the SR240A preamplifier with a 50 Ω load at the input instead of the signal in two frequency ranges: (a) 7–400 Hz in 0.2 Hz steps and (b) 10–2000 Hz in 1 Hz steps, obtained by fast Fourier transform of an oscillogram measured in time windows of 1 or 5 s, respectively.

5. Time Profiles of the NovoFEL Macropulses Obtained with Different Detectors

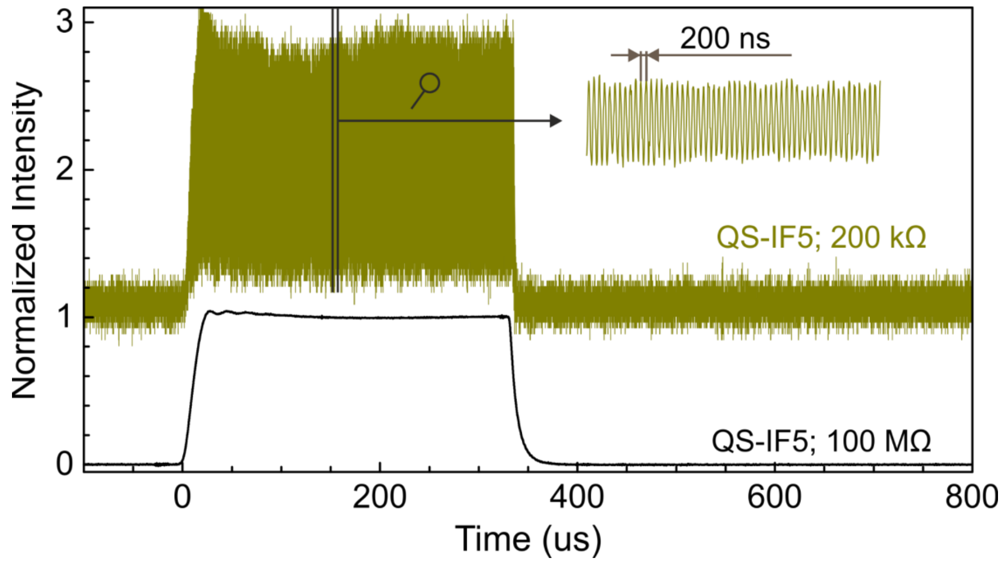

Figure S15. Time profiles of the NovoFEL macropulses of 350 μs duration obtained at 66.7 cm^{-1} (150 μm; 2.0 THz) using the QS-IF5 pyroelectric detector with 2 different resistances in the preamplifier feedback: 200 kΩ (dark green, vertically shifted) and 100 MΩ (black). The fine structure of the NovoFEL macropulses is visible with the 200 kΩ resistance.

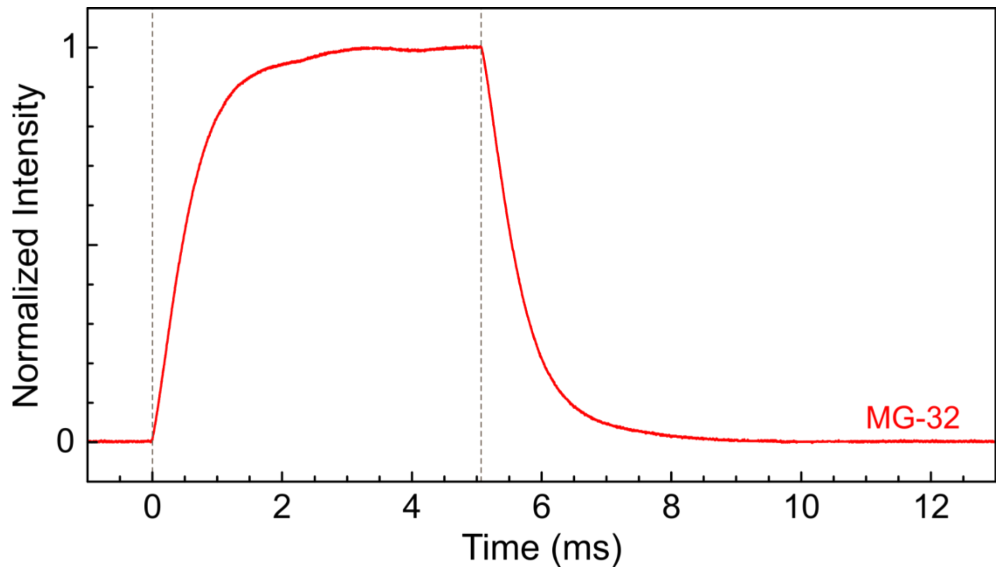

Figure S16. Time profiles of the NovoFEL macropulses of 5 ms duration obtained at 66.7 cm^{-1} (150 μm; 2.0 THz) using the MG-32 pyroelectric detector.

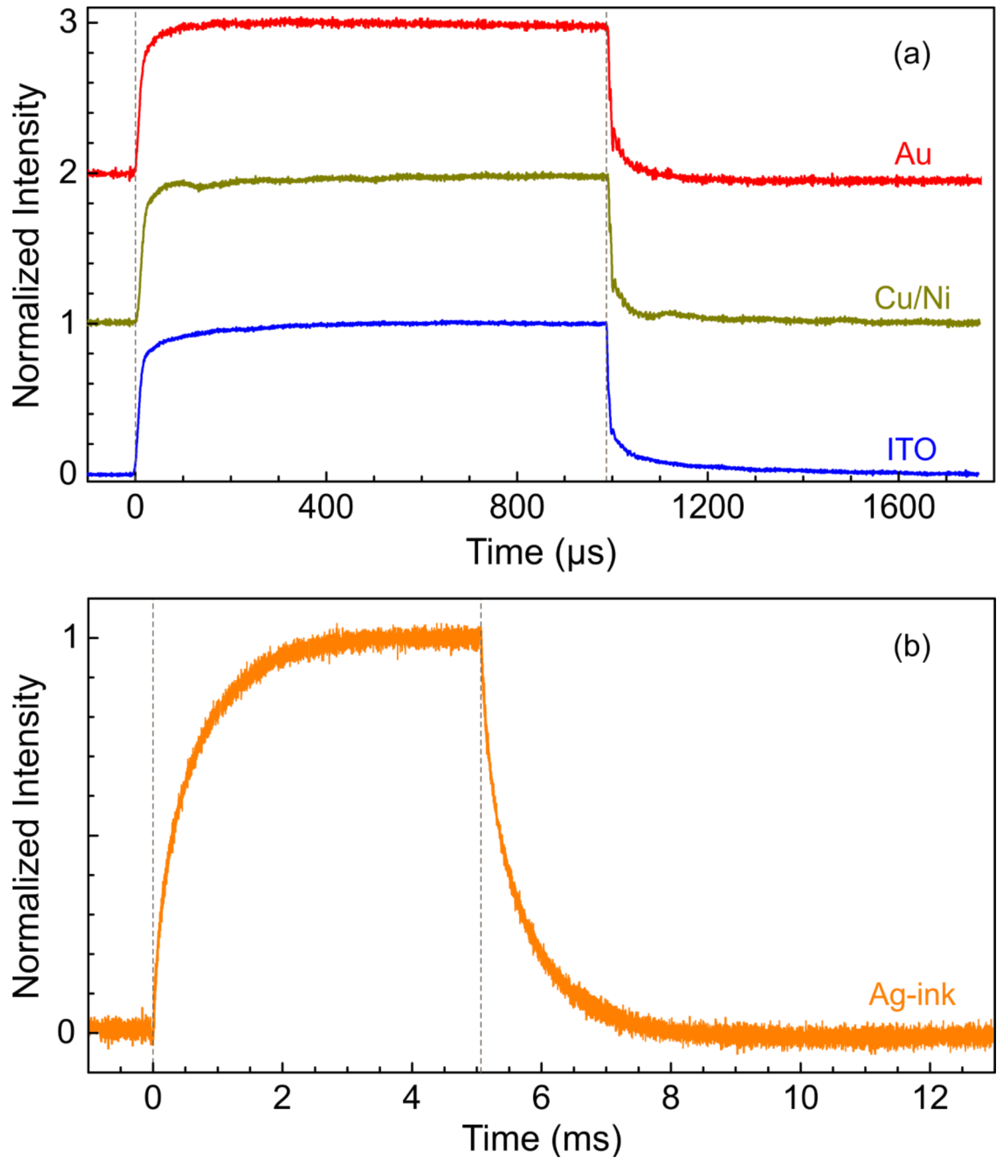

Figure S17. (a) Time profiles of the NovoFEL macropulses of 1 ms duration obtained at 66.7 cm⁻¹ (150 μm; 2.0 THz) using different PVDF-based pyroelectric detectors: ITO (blue); Cu/Ni (dark green); Au (red). Each subsequent profile is vertically shifted. (b) Same as (a) for a 5 ms macropulse measured with the Ag-ink pyroelectric detector.
